# Periodic fasting induced reconstitution of metabolic flexibility improves albuminuria in patients with type 2 diabetes

**DOI:** 10.1016/j.molmet.2025.102257

**Published:** 2025-09-24

**Authors:** Alba Sulaj, Phong B.H. Nguyen, Gernot Poschet, Elisabeth Kliemank, Thomas Fleming, Lea Henke, Wiebke Neibig, Stefan Kopf, Rüdiger Hell, Valter D. Longo, Stephan Herzig, Peter P. Nawroth, Michael P. Menden, Julia Szendroedi

**Affiliations:** 1 Department of Endocrinology, Diabetology, Metabolic Diseases and Clinical Chemistry (Internal Medicine I), University Hospital of Heidelberg, Heidelberg, Germany; 2 German Center for Diabetes Research (DZD), Neuherberg, Germany; 3 University of Luebeck and University Hospital of Schleswig-Holstein (UKSH) Luebeck, Germany; 4 Computational Health Center, Institute of Computational Biology, Helmholtz Munich, Munich, Germany; 5 Department of Biology, Ludwig-Maximilians University, Munich, Germany; 6 Centre for Organismal Studies (COS), Metabolomics Core Technology Platform, University of Heidelberg, Heidelberg, Germany; 7 Longevity Institute, School of Gerontology, and Department of Biological Sciences, University of Southern California, Los Angeles, CA, United States; 8 FIRC Institute of Molecular Oncology, Italian Foundation for Cancer Research Institute of Molecular Oncology, Milan, Italy; 9 Institute for Diabetes and Cancer, Helmholtz Center Munich, Neuherberg, Germany; 10 Joint Heidelberg-IDC Translational Diabetes Program, Internal Medicine I, Heidelberg University Hospital, Heidelberg, Germany; 11 Chair Molecular Metabolic Control, Technical University Munich, Munich, Germany; 12 Joint Heidelberg-IDC Translational Diabetes Program, Helmholtz Center Munich, Neuherberg, Germany; 13 Department of Biochemistry and Pharmacology, Bio21 Molecular Science and Biotechnology Institute, The University of Melbourne, Parkville, Victoria, Australia

**Keywords:** Periodic fasting, Metabolic flexibility, Albuminuria, Type 2 diabetes

## Abstract

**Objective:**

Metabolic inflexibility has been shown to be associated with type 2 diabetes (T2D) and diabetic nephropathy (DN). However, data are lacking, proving that reconstitution of metabolic flexibility by using a 6-month periodic fasting (PF) regimen may improve albuminuria.

**Methods:**

In this post hoc analysis of a randomized-controlled trial, we investigated whether the PF regimen enhanced metabolic flexibility in individuals with T2D and DN showing improvement of albuminuria (responders) compared to non-responders. Participants followed every month either a 5-day fasting-mimicking diet or a Mediterranean diet for 6 months. LC-MS/MS-based comprehensive metabolic profiling was performed in plasma samples before, during, and after the intervention. Changes in metabolomic patterns and enriched signalling pathways were analysed between study groups.

**Results:**

PF induced a sustained shift toward enhanced fatty acid oxidation, lipid utilization, and amino acids turnover, particularly in responders. Responders exhibited persistent elevations in short-chain acylcarnitines and cholesteryl esters, indicating more efficient lipid oxidation and tighter integration of lipid metabolism with the tricarboxylic acid cycle. Increased glycine and serine levels suggested enhanced cellular maintenance, a protein-sparing effect, and a metabolic shift favouring lipid over carbohydrate. In contrast, non-responders demonstrated only transient and limited metabolic shifts. Unsupervised clustering identified distinct metabolic response patterns, reinforcing the potential of personalized dietary interventions.

**Conclusions:**

These findings demonstrate that diet-induced restoration of metabolic flexibility is associated with improved albuminuria in T2D, suggesting broader implications for precise nutritional strategies in diabetes management.

## Highlights

• Metabolic inflexibility and mitochondrial dysfunction are hallmarks of type 2 diabetes (T2D) and contribute to the development of complications such as diabetic nephropathy (DN).
• Periodic fasting (PF) effectively restored metabolic flexibility in individuals with T2D and DN.
• PF induced a sustained shift toward enhanced fatty acid oxidation, lipid utilization, and amino acids turnover, particularly in individuals with improved albuminuria.
• Diet-induced restoration of metabolic flexibility was associated with improved albuminuria in T2D, suggesting broader implications for precise nutritional strategies in diabetes management.

## 1 Introduction

A precision diabetology approach – classifying diabetes and prediabetes into distinct subphenotypes – has emerged as a promising strategy for tailoring dietary and therapeutic interventions [[1], [2], [3], [4]]. Advancing this approach requires a deeper understanding of the pathophysiological mechanisms driving metabolic heterogeneity in diabetes [5]. A central mechanism potentially driving this heterogeneity is metabolic flexibility, the capacity of an organism to efficiently shift between different energy substrates, such as glucose and fatty acids, depending on nutrient availability and energy demands. Metabolic inflexibility and mitochondrial dysfunction are hallmarks of type 2 diabetes (T2D) and contribute to the development of complications such as diabetic nephropathy (DN) [6,7].

Our previous work demonstrated that periodic fasting (PF) – implemented as a monthly fasting-mimicking diet (FMD) over six months – improved metabolic parameters and DN in individuals with T2D and microalbuminuria, without impairing nerve function [8,9]. Furthermore, glucose intake during the refeeding phase after fasting elicited heterogeneous oxidative stress responses, depending on the presence of diabetes complications [10]. While these findings highlighted the potential benefits of PF in specific subgroups, the underlying mechanisms, particularly how PF may restore metabolic flexibility, and its associated metabolic responses remain poorly understood.

Impaired metabolic flexibility and mitochondrial stress promote lipid accumulation, oxidative damage, and inflammation in renal tissues [6,11,12]. Preclinical studies indicate that fasting regimens may counteract these processes by enhancing fatty acid oxidation, ketogenesis, mitochondrial efficiency, and autophagy [13] - processes collectively representing improved metabolic flexibility. In rodent models of DN, intermittent fasting reduced lipid deposition, improved mitochondrial integrity, and delayed kidney injury [[14], [15], [16]]. While these findings are promising, clinical evidence remains sparse. Studies establishing causality between restored metabolic flexibility and clinical improvement in individuals with T2D are lacking. Thus, it remains unclear whether fasting can reliably induce similar metabolic adaptations in humans with T2D and kidney disease, and whether such adaptations translate into improved renal outcomes. Our study addresses this gap by linking metabolic shifts to kidney function in a clinical trial setting.

Nutritional and dietary interventions regulate key metabolic pathways, including glucose homeostasis, insulin sensitivity, inflammation, oxidative stress, and immune responses [[17], [18], [19]]. Traditionally, these strategies have focused on managing obesity and cardiometabolic health through caloric restriction, macronutrient modifications, fasting regimens, and time-restricted eating protocols [17]. Beyond metabolic diseases, emerging evidence suggests that such dietary approaches may confer benefits in conditions such as cancer, neurodegenerative disorders, and autoimmune diseases [[20], [21], [22]]. However, interindividual variability in response to dietary interventions is increasingly recognized, with some individuals experiencing adverse outcomes, including frailty, sarcopenia and increased health risks [[23], [24], [25], [26], [27]]. For instance, an analysis of the Action for Health in Diabetes (Look AHEAD) trial revealed that an intensive lifestyle intervention - combining caloric restriction and increased physical activity - was paradoxically associated with a higher risk of cardiovascular events in individuals with type 2 diabetes (T2D) and poor glycemic control [24]. Similarly, dietary composition plays a critical role beyond caloric intake alone. Low-carbohydrate diets have been associated with higher mortality, potentially due to elevated animal protein intake [25], whereas low-protein diets have been associated with higher mortality in individuals over 65 years, likely due to sarcopenia [27]. These findings underscore the necessity for individualized dietary interventions that consider baseline metabolic characteristics, such as metabolic flexibility, in order to optimize benefits while minimizing risks.

In this study, we conducted a post hoc analysis of a randomized clinical trial to investigate the systemic metabolic effects of PF in individuals with T2D and DN. Using longitudinal metabolic profiling, we aimed to characterize adaptive metabolic shifts and identify predictors of favorable responses to PF. We compared these metabolic adaptations to those observed with a Mediterranean diet. We aimed to (i) characterize the metabolic flexibility induced by PF in individuals with T2D and DN, (ii) identify metabolic biomarkers predicting favorable responses to PF, particularly improved albuminuria, and (iii) to compare the metabolic adaptations elicited by PF with those observed in a mediterranean diet, highlighting potential mechanisms through which dietary interventions influence kidney function and metabolic health in diabetes. With this study we wanted to provide mechanistic insights into the role of metabolic flexibility in dietary response heterogeneity, highlighting the potential of tailored dietary strategies in T2D management.

## 2 Methods

### 2.1 Study participants

The initial randomized controlled clinical study was conducted as a proof-of-concept to assess the feasibility, safety, and potential efficacy of the intervention. The study was conducted at the Clinic of Endocrinology, Diabetology, Metabolic Diseases and Clinical Chemistry at the University Hospital of Heidelberg, Germany. The study protocol was approved by the ethics committee of the University of Heidelberg (*Ethic-Nr. S-459/2018*) in compliance with national guidelines and the Declaration of Helsinki. The study was registered at the German Clinical Trials Register (Deutsches Register Klinischer Studien *DRKS-ID:* DRKS00014287), and all participants provided written informed consent. The primary outcome of the study compared albuminuria between the two dietary interventions, with results previously reported [8]. An exploratory aim, which is the focus of this manuscript, was to compare metabolite profiles between the study diets. Detailed information on the study participants, inclusion and exclusion criteria, and study design have been previously published [8]. In brief, the study included female and male individuals with T2D and diabetic nephropathy, aged 50–75 years, non-smokers, with a BMI between 23 and 40 kg/m^2^, and without severe cardiovascular, renal, hematological, liver, peripheral artery diseases, or a history of cancer in the 5 years prior to the study. Participants were randomized (1:1) using a stratified computed procedure (Randomizer Version 2.0.3© Institute for Medical Informatics, Statistics and Documentation, Medical University of Graz) based on BMI (cutoff: 30.0 kg/m^2^) and hemoglobin A1c (cutoff: 7.0%) into either periodic fasting or Mediterranean diet group. Participants were instructed to follow either a fasting-mimicking diet or a Mediterranean diet for 5 consecutive days each month, returning to their normal diet until the next cycle, which began approximately 25 days later. The total study duration was 6 months. For this post hos analysis, we included only participants who completed the 3-month dietary intervention to ensure a complete dataset and robust analysis of metabolites. As previously reported, this resulted in a total of 38 individuals [8] whose plasma samples were available for analysis: 18 from the Mediterranean diet group (5 female/13 male) and 20 from the fasting group (6 female/14 male). All participants completed the 3-month diet intervention, while the 6-month intervention was completed from 16 in the Mediterranean diet group (5 female/11 male) and 17 in the fasting group (5 female/12 male). Fourteen participants in the Mediterranean diet group completed the follow-up (5 female/9 male) and 15 in the fasting diet group (4 female/11 male in the fasting group). Due to small sample size, sex differences were not analyzed. Considering the exploratory nature of this post hoc analysis, we applied a ≥10% reduction in albuminuria as the threshold to characterize treatment response. In the original trial, a 30% reduction in albuminuria was predefined for the power analysis [8], as this threshold is commonly used as a clinically meaningful benchmark associated with improved renal outcomes [28,29]. Reporting treatment effects below the 30% threshold is common in intervention studies focusing on albuminuria, particularly in early or subgroup exploratory analyses [30].

There was no broad consent for this study, therefore the metabolomics data and metadata cannot be made freely available in a public repository. However, the data sets generated from the study are available from the corresponding author on reasonable request.

### 2.2 Dietary intervention

The fasting-mimicking diet used in this study was a plant-based diet designed to mimic fasting effects on glucose and ketone bodies. It provided a daily intake of 4,600 kJ (11% protein, 46% fat, and 43% carbohydrates) on day 1 and 3,000 kJ (9% protein, 44% fat, and 47% carbohydrate) from day 2–5 [31]. The Mediterranean diet maintained participants' usual caloric intake before the study, and participants were instructed to adhere to the criteria outlined in the Mediterranean diet score [32]. Participants on insulin therapy were instructed to discontinue short-acting insulin and reduce long-acting insulin by 50% during fasting. Oral antidiabetic therapy was also halted during the fasting periods. Glycaemic levels were self-monitored, with participants measuring fasting and 2-hour postprandial levels at least four times daily using a capillary blood glucose monitoring system (Accu-Check Guide®, Roche). A 24-hour telephone platform was available for participants to report any hypoglycemic or hyperglycemic episodes. All participants were advised to avoid excessive physical activity during the diet intervention. Safety was monitored through assessment of vital signs, physical examination, recording of adverse events following the Common Terminology Criteria for Adverse Events, and laboratory results at each visit.

### 2.3 Sample size

The original study was adequately powered to evaluate the primary outcome, as previously reported [8]. The metabolome analysis presented here was exploratory, with no predetermined sample size. Due to the study’s design, the research team was not blinded to allocation during the intervention and outcome assessment.

### 2.4 Blood sample collection and chemistry

Blood samples were collected at baseline, immediately after the 3rd and 6th diet cycles, and again 3 months following the 6-month intervention. Samples were drawn in an overnight fasting state and processed immediately in the Central Laboratory of the University Hospital of Heidelberg under standardized conditions. Beta-hydroxybutyrate levels were measured at each visit using venous blood (StatStrip® Glucose/Ketone Meter System, Nova® Biomedical).

### 2.5 Metabolomic sample collection

Targeted metabolomics analysis was performed on stored plasma samples (kept at −80 °C since collection) from 38 participants, covering all study time points as written above for a total of 140 samples). All samples were processed in a single batch following the manufacturer’s protocol for the MxP® Quant 500 kit (Biocrates). Briefly, 10 μL of each sample or blanks was pipetted onto a 96 well-plate containing calibrators and internal standards using an automated liquid handling station (epMotion 5075, Eppendorf), and then dried under a nitrogen stream using a positive pressure manifold (Waters). Following this, 50 μL of phenyl isothiocyanate 5% (PITC) was added to each well to derivatize amino acids and biogenic amines. After an incubation period of 1 h at room temperature, the plate was dried again. To resolve all extracted metabolites, 300 μL of 5 mM ammonium acetate in methanol was added to each filter and incubated for 30 min. The extract was then eluted into a new 96-well plate using positive pressure. For the LC-MS/MS analyses, 150 μL of the extract was diluted with an equal volume of water. Similarly, for FIA-MS/MS analyses, 10 μL of the extract was diluted with 490 μL of FIA solvent provided by Biocrates. Following dilution, LC-MS/MS and FIA-MS/MS measurements were conducted in both positive and negative mode. Chromatographic separation was achieved using an UPLC I-class PLUS (Waters) system coupled with a SCIEX QTRAP 6500+ mass spectrometry system in electrospray ionization (ESI) mode. The LC gradient composition and specific 50 × 2.1 mm column were provided by Biocrates. Data were recorded using the Analyst (Version 1.7.2 Sciex) software suite and further processed with MetIDQ software (Oxygen-DB110-3005). All metabolites were identified using isotopically labeled internal standards and multiple reaction monitoring (MRM) with optimized MS conditions provided by Biocrates. For quantification, either a seven-point calibration curve or one-point calibration was used, depending on the metabolite class. FIA-MS/MS analysis does not provide specific information regarding either the positions or the chain lengths of the fatty acid residues linked to each lipid’s backbone. As a consequence, the detected signal is a sum of several isobaric/isomeric lipids or acylcarnitines.

### 2.6 Data processing and analyses

#### 2.6.1 Validation and filtering

Data validation and quantification were performed using MetIDQ (Oxygen-DB110-3005). Here, metabolites were further categorized based on their quantitation ranges. Additional filtering per metabolite was based on the limit of detection (LOD), limit of quantification (LOQ) and concentration within the quantitative range (valid). Before downstream analyses, the metabolite values that fell outside of the LOD and LOQ were marked as missing, and all the metabolites and samples that had missing rates higher than 30% were removed. The samples that did not have record in all time points were also filtered out. The remaining missing data were imputed using the k-nearest neighbor algorithm. The laboratory and physiological data were also processed in the same manner.

#### 2.6.2 Longitudinal differential expression analysis

We selected samples with complete clinical and metabolomic data across timepoints, after data processing as described above, yielding 15 controls, 10 responders and 6 non-responders. Using the R package limma [33], we analyzed metabolic changes within each diet group by comparing the log-transformed metabolite levels at 3-month, 6-month and follow-up time points to baseline, adjusting for age, sex and diabetes duration. Multiple hypothesis testing corrections were employed using the Benjamini-Hochberg method [34].

#### 2.6.3 Metabolite set enrichment analysis

We conducted metabolite set enrichment analysis to functionally interpret the identified metabolic changes. Using the R package *fgsea* [35] and reference metabolite sets obtained from the *Reactome* database, we applied gene set enrichment analysis (GSEA) [36] to rank metabolites based on t-statistics derived from the differential expression analysis. For each metabolite set, an enrichment score (ES) utilizing Kolmogorov–Smirnov statistics, indicated the set’s up- or downregulated relative to the phenotype. Statistical significance of the ES score was determined by permutation testing against a null distribution, with resulting p-values adjusted using Benjamini-Hochberg correction.

#### 2.6.4 Unsupervised clustering of patients using longitudinal clinical information

After data processing we obtained 31 samples and 54 clinical variables including demographics, physical measurements and lab tests (Supplementary Table S3). Manhattan distance matrix for each variable was averaged to create an aggregated distance matrix for consensus clustering K-means clustering was performed with k ranging from 2 to 6, selecting the optimal k by examining sample distribution in consensus matrices. Clustering was conducted using the R package *ConsensusClusterPlus* [37].

## 3 Results

### 3.1 Experimental design and analysis workflow

**Figure 1:**
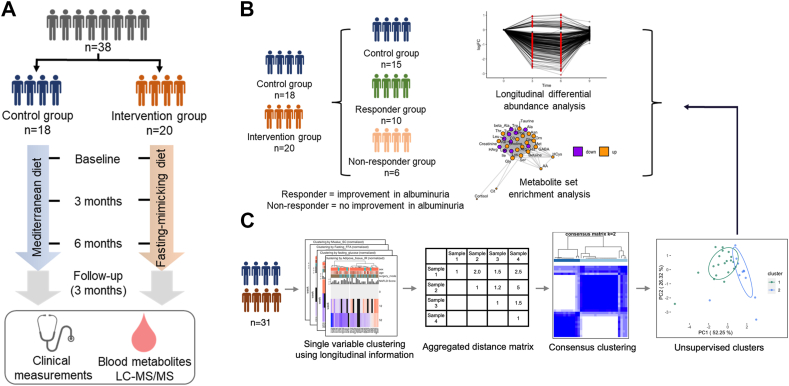
**Experimental Design and Analysis Workflow.** A) Overview of the study design and dataset. B) Clustering based on albuminuria response, followed by metabolomic analysis of the study groups. C) Unsupervised clustering of all participants using longitudinal clinical information, followed by metabolomic analysis of the identified subgroups.

The study included 38 participants, with 18 in the control group (Mediterranean diet, MD) and 20 in the treatment group (fasting-mimicking diet, FMD). Participants were instructed to follow either FMD or MD for 5 consecutive days each month, for a total study duration of 6 months. Clinical data and blood samples were collected at fasted conditions at baseline, directly after each 5-day dietary cycle at months 3 and 6, and again 3 months post-intervention, enabling analysis of both short-term and long-term metabolic effects. The design and analysis workflow are illustrated in Figure 1A, with baseline characteristics summarized in Supplementary Table 1.

After applying data quality-control filtering, 15 samples from the control group and 16 samples from the intervention group (a total of 31 samples) were included in the analysis of metabolic responses. We conducted longitudinal differential abundance analysis and metabolite set enrichment analysis, identifying key metabolites and pathways modulated by each intervention (Figure 1B). In the FMD group, participants were further classified as responders or non-responders based on urinary albumin-to-creatinine ratio (uACR) improvement, enabling a comparative analysis of metabolic adaptations (Figure 1B). Among responders, one participant showed a 10% reduction in albuminuria, while the other responders improved by ≥ 30%. Additionally, we applied single-variable clustering to longitudinal clinical data to generate an aggregated distance matrix, which was subsequently subjected to consensus clustering to robustly define participant subgroups. This approach identified two distinct metabolic response patterns, independent of intervention groups, highlighting heterogeneous metabolic adaptations within both the FMD and MD cohorts (Figure 1C).

### 3.2 Dynamic metabolic response to fasting-mimicking diet intervention

**Figure 2:**
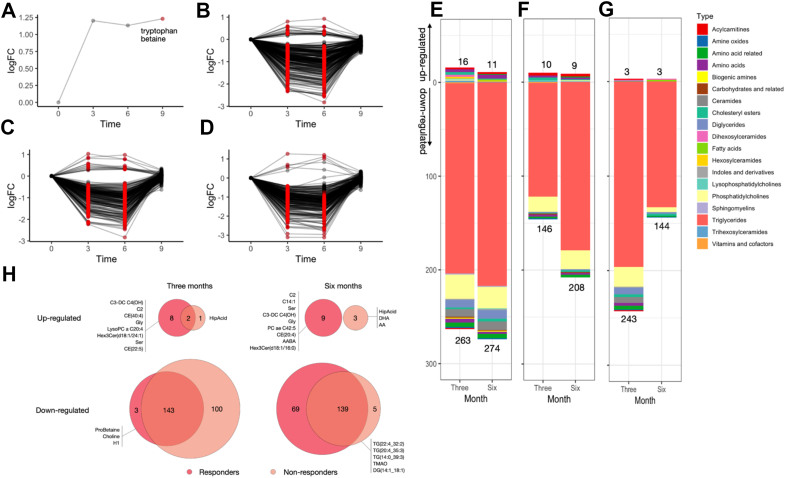
**Changes in metabolites during the study.** Longitudinal metabolomic changes in different study groups including A) control group (Mediterranean diet), B) intervention group (fasting-mimicking diet), C) responder group, and D) non-responder group. LogFC is the log-transformation of the fold change. Significantly differential metabolites are marked in red. Total number of metabolites and metabolite categories that changed significantly after 3 and 6 months of fasting-mimicking diet in E) the intervention group, F) responder group, and G) non-responder group. H) Number of significant metabolites that are overlapped and unique for responder and non-responder group after three months and six months. Abbreviations: C3-DC (C4–OH) hydroxybutyrylcarnitine, C2 acetylcarnitine, CE(20:4) and CE(22:5) cholesteryl esters, lysoPC a C20:4 long-chain lysophosphatidylcholine, Hex3Cer(d18:1/24:1) trihexosylceramide, Gly glycine, Ser serine, HippAcid hippuric acid, Probetaine proline betaine, H1 hexoses, C14:1 tetradecenoylcarnitine, PC ae C42:5 phosphatidylcholine, CE(20:4) cholesteryl ester, AABA α-aminobutyric acid, Hex3Cer(d18:1/16:0) trihexosylceramide, DHA docosahexaenoid acid, AA arachidonic acid, TG(22:4_32:2), TG(20:4_35:3) and TG(14:0_39:3) triglycerides, DG(14:1_18:1) diglyceride and TMAO trimethylamine N-oxide. (For interpretation of the references to colour in this figure legend, the reader is referred to the Web version of this article.)

While baseline metabolite levels did not differ between study groups (Supplementary Fig. 1), significant longitudinal changes within each group were observed in the MD and FMD groups. Minimal changes were observed in the MD group, with tryptophan betaine as the only metabolite increasing at follow-up (adjusted *p*-value = 0.04; Figure 2A). Tryptophan betaine, a derivative of tryptophan metabolism, has been implicated in gut microbiota modulation and metabolic health [38,39]. In contrast, the FMD group exhibited substantial metabolic shifts at both three and six months, with most metabolites returning to baseline post-intervention (Figure 2B). A total of 302 metabolites were significantly altered at one or more time points relative to baseline. By the month 3, 279 metabolites were affected, with 263 being downregulated and 16 upregulated. At six months, 285 metabolites were altered (274 downregulated and 11 upregulated; adjusted *p*-value <0.05; Figure 2B,E). Additionally, 262 metabolites showed sustained changes at both 3 and 6 months, (254 downregulated, 8 upregulated), indicating a persistent metabolic response to FMD.

Downregulated metabolites were primarily triglycerides, diglycerides and phosphatidylcholines, suggesting reduced circulating lipid levels, likely due to enhanced lipid utilization or redistribution. Meanwhile, acylcarnitines, amino acids, and cholesteryl esters, were upregulated, reflecting increased lipid oxidation, gluconeogenesis-derived amino acid turnover and maintenance of cellular homeostasis by fatty acid sequestration (Figure 2E). These findings indicate that FMD promotes a shift toward stored energy utilization, enhancing metabolic flexibility.

Among detectable acylcarnitines, key metabolites included carnitine (C0), acetylcarnitine (C2), propionylcarnitine (C3), hydroxybutyrylcarnitine (C4–OH) and tetradecenoylcarnitine (C14). In the FMD group, acetylcarnitine (C2), hydroxybutyrylcarnitine (C4–OH) and tetradecenoylcarnitine (C14:1) significantly increased, while propionylcarnitine (C3) significantly decreased at 3 months. The increase in acetylcarnitine (C2) and tetradecenoylcarnitine (C13), and the decrease in propionylcarnitine (C3) persisted at 6 months (Figure 2E).

Amino acid alterations were more pronounced in the FMD group, which exhibited increased glycine and decreased levels of glutamine, alanine, valine and proline after three months (Supplementary Fig. 2). At six months, additional increases in methionine, serine, and threonine levels were observed, alongside further decreases in glutamate, tyrosine, and proline (Supplementary Fig. 3). At follow-up, metabolite levels returned to baseline in both groups (Figure 2), with threonine remaining elevated in the FMD group relative to the MD group (Supplementary Fig. 4). This consistent pattern of metabolite alterations between the third and sixth months suggests sustained metabolic effects throughout the intervention period.

Notably, only 23 metabolites changed exclusively at the six-month mark, with α-aminobutyric acid, arachidonic acid, and methionine upregulated, while key lipid classes, including triglycerides, diglycerides, phosphatidylcholines, lysophosphatidylcholines, ceramides, and cholesteryl ester CE (17:1), were downregulated (Figure 2B,E). This specific pattern suggests an intensified lipid catabolism and mobilization phase towards the end of the intervention, potentially marking peak metabolic adaptation to FMD.

### 3.3 Distinct metabolic adaptations in FMD responders versus non-responders

We have previously shown that FMD improves albuminuria in some participants (*responders*), while others do not experience this effect (*non-responders*) [8]. This differential response provides an opportunity to investigate FMD-dependent metabolic pathways that may underlie its effectiveness in some individuals but not others. We focused on the metabolic profiles of FMD group participants identified as either responders (*n* = 11, 55%) or non-responders (*n* = 9, 45%) (Supplementary Table 2) and conducted the same metabolomic analyses within each subgroup (Figure 1B). In the MD group, 8 participants (44%) showed an improvement in albuminuria levels, while 10 participants (56%) did not. Given that within-individual albuminuria variability in T2D is approximately 49%, the higher proportion of responders in the FMD group may indicate a true intervention effect beyond random variation [40].

In the responder group, 224 metabolites showed significant alterations at least once between the third and sixth months relative to baseline, with 156 changes (146 downregulated, 10 upregulated) by the third month and increasing to 217 changes (208 downregulated, 9 upregulated) by the sixth month (Figure 2C,F). In contrast, non-responders exhibited a distinct pattern, with 249 metabolites altered at least once during the study period, with an early shift of 246 changes (243 downregulated, 3 upregulated) by the third month, which decreased to 147 altered metabolites (144 downregulated, 3 upregulated) by the sixth month (Figure 2D,G). By the follow-up period, metabolite levels in both groups had returned to baseline, suggesting that the FMD-induced changes were transient and differed in timing and extent between responders and non-responders.

Responders exhibited greater and sustained downregulation of triglycerides and phosphatidylcholines after six months (Figure 2F), while non-responders showed similar changes much earlier—by the third month (Figure 2G). Additionally, certain metabolite groups, including acylcarnitines, amino acids, and cholesteryl esters, were exclusively upregulated in responders (Figure 2F). In contrast, non-responders showed minimal upregulation of three metabolites (hippuric acid, docosahexaenoid acid and arachidonic acid) (Figure 2F), suggesting a more limited metabolic response to FMD.

Among the 11 upregulated metabolites in the FMD group at the third month, 8 were unique to responders—such as hydroxybutyrylcarnitine (C4–OH), acetylcarnitine (C2), cholesteryl esters CE(20:4) and CE(22:5), lysophosphatidylcholine lysoPC a C20:4, trihexosylceramide Hex3Cer(d18:1/24:1), glycine, and serine—while only 1 metabolite, hippuric acid, was unique to non-responders (Figure 2H). Downregulated metabolites were also more extensive in non-responders, where 100 metabolites (mainly triglycerides and phosphatidylcholines) were reduced, suggesting an initial lipid depletion phase that may not sustain long-term mobilization (Figure 2H). Proline betaine, choline and hexoses were reduced in responders. By the sixth month, responders continued to upregulate metabolites linked to lipid oxidation and amino acid turnover, such as acetylcarnitine (C2), tetradecenoylcarnitine (C14:1), hydroxybutyrylcarnitine (C4–OH), phosphatidylcholine ae C42:5, cholesteryl esters CE(20:4), α-aminobutyric acid, trihexosylceramide Hex3Cer(d18:1/16:0), serine and glycine. Non-responders instead upregulated hippuric acid, docosahexaenoid acid and arachidonic acid, potentially reflecting an alternative lipid handling response (Figure 2H). From the downregulated metabolites, only 5 metabolites changed in the non-responder group (triglycerides TG(22:4_32:2), TG(20:4_35:3) and TG(14:0_39:3), diglyceride DG(14:1_18:1) and trimethylamine N-oxide) (Figure 2H).

When analyzing acylcarnitine ratios, the FMD group showed a significant increase in the acetylcarnitine/carnitine ratio (C2/C0) compared to the MD group, with no difference between responders and non-responders (Supplementary Fig. 5a). Similarly, the propionylcarnitine/acetylcarnitine ratio (C3/C2) decreased significantly in both FMD responders and non-responders compared to the MD group (Supplementary Fig. 5b).

When analyzing responder vs. non-responder of the MD group included in the longitudinal differential expression analysis, we found no difference in metabolite levels during the study (Supplementary Fig. 6a and 6b).

### 3.4 Differential lipid and protein pathways in FMD responders and non-responders

**Figure 3:**
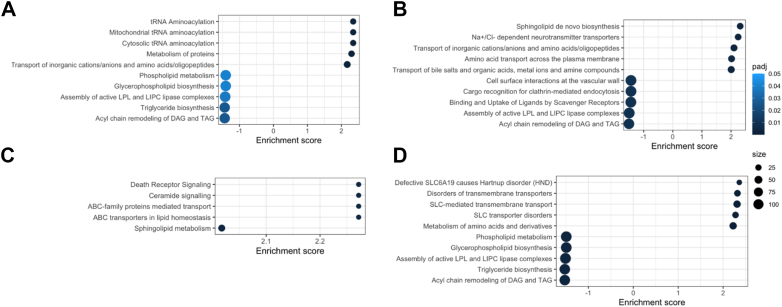
**Metabolite enrichment pathway analysis.** Here, highlighted are the top 5 upregulated and top 5 downregulated pathways associated with A) responder group - 3 month diet intervention, B) responder group - 6 month diet intervention, C) non responder group - 3 month diet intervention, and D) non responder group - 6 month diet intervention.

Metabolite enrichment analysis showed that both responders and non-responders experienced alterations in lipid-related pathways, though with different timing and extent. In responders, five pathways—including phospholipid metabolism, glycerophospholipid biosynthesis, and triglyceride biosynthesis—were downregulated by the third month, whereas non-responders showed downregulation of these same pathways only by the sixth month (Figure 3A,D). Additionally, in responders, two key pathways (lipoprotein and hepatic lipase complex assembly, and acyl chain remodelling) were upregulated by six months, potentially reflecting a progressive shift towards enhanced lipid mobilization and utilization. (Figure 3C). Notably, unique pathways, including cell surface interactions and clathrin-mediated endocytosis, were downregulated exclusively in responders at six months, suggesting a more adaptable cellular response over time, while no significant downregulation was observed in non-responders at three months (Figure 3C).

**Figure 4:**
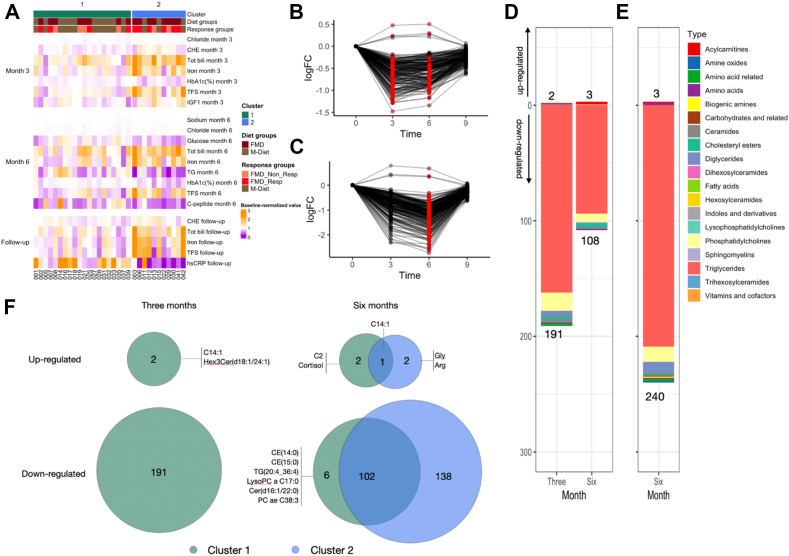
**Unsupervised clustering revealed distinct metabolic clusters.** A) Clinical variables that are significantly different among the groups, normalized for baseline level. B-C) Changes in metabolites that are significantly expressed at least one time point compared to baseline for cluster 1 and 2, respectively. Total number of metabolites that changed significantly after 3 and 6 months of diet intervention in D) cluster 1, and E) cluster 2. F) Number of significant metabolites that are overlapped and unique for cluster 1 and cluster 2 after three months and six months. Abbreviations: CHE cholinesterase, Tot bili total bilirubin, TFS transferrin saturation, IGF1 insulin-like growth factor 1, TG triglycerides, hsCRP high-sensitivity CRP, C14:1 tetradecenoylcarnitine, Hex3Cer(d18:1/24:1) trihexosylceramide, C2 acetylcarnitine, Gly glycine, Ser serine, CE(20:4) and CE(22:5) cholesteryl esters, lysoPC a C20:4 lysophosphatidylcholine, Cer(d16:1/22:0) ceramide, PC ae C38:3 phosphatidylcholine.

Upregulation patterns also differed between the groups: responders showed increased activity in pathways related to protein synthesis and amino acid transport, aligning with their enhanced metabolic flexibility observed earlier, such as greater amino acid mobilization and lipid oxidation (Figure 3A,B). In contrast, non-responders showed upregulation of pathways related to ceramide signaling and lipid transport, perhaps reflecting a less efficient or alternative lipid handling strategy (Figure 3, Figure 4C and 4D). These results indicate that responders exhibit a sustained shift in key metabolites related to lipid oxidation and amino acid turnover, consistent with enhanced metabolic flexibility, while non-responders show an early but less durable adaptation.

### 3.5 Unsupervised cluster analysis distinguishes metabolic profiles and flexibility in FMD responders

Unsupervised clustering of clinical data grouped participants into two clusters: Cluster 1, which primarily consisted of more MD participants and FMD non-responders (12 MD, 8 FMD; 4 responders and 4 non-responders), and Cluster 2, which was predominantly composed of FMD responders (3 MD, 8 FMD; 6 responders and 2 non-responders). Cluster 2 showed improvements in glycaemic control, HbA1c, fasting glucose, c-peptide, triglycerides, and high sensitivity C-reactive protein (hsCRP), along with increased insulin-like growth factor-1 (IGF1), aligning with a more robust FMD adaptation. Cluster 1, in contrast, showed reduction in cholinesterase, iron, transferrin saturation, total bilirubin, chloride, and sodium, indicating a different or less targeted metabolic adjustment (Figure 4A–C; Supplementary Table 3, Supplementary Fig. 7a–u).

Metabolically, Cluster 1, primarily comprising MD participants and FMD non-responders, showed early lipid utilisation by the third month, with significant alterations in 203 metabolites by the third or sixth months, with 193 metabolites changed in the third month and 111 by the sixth month (Figure 4B,D). Notably, most of these metabolites were downregulated (mostly triglycerides, diglycerides and phosphatidylcholines), with peak changes occurring by the third month and returning to baseline levels by the follow-up (Figure 4B).

Cluster 2 (mainly FMD responders) showed a delayed yet more sustained metabolic shift compared to Cluster 1, with significant alterations in 243 metabolites by the sixth month, with 240 downregulated, mostly being triglycerides, diglycerides and phosphatidylcholines (Figure 4C,E). Unique increases were observed in tetradecenoylcarnitine (C14:1) and trihexosylceramide in Cluster 1 at three months, while Cluster 2 displayed increases in glycine and arginine only after six months (Figure 4E,F). Thus, cluster 2 participants showed robust metabolic improvements and sustained shifts in key pathways, suggesting a more flexible metabolic phenotype.

Metabolite enrichment analysis of Cluster 1 and Cluster 2 revealed similar changes as in the non-responder group and the responder group, respectively. Most downregulated pathways were similar in both clusters and timepoints of the study: 1. cell surface interactions at the vascular wall, 2. cargo recognition for clathrin-mediated endocytosis, 3. assembly of active lipoprotein lipase and hepatic triacylglycerol lipase complexes and 4. acyl chain remodeling of diacylglycerol and triacylglycerol (Supplementary Figure 8a-8d). Cluster 2, similar to responders, upregulated pathways related to protein synthesis and amino acid metabolism, indicative of enhanced metabolic flexibility compared to Cluster 1 (Supplementary Figure 8c and 8d). Cluster 1, similar to non-responders, upregulated pathways in ceramide signaling and lipid transport, but lacked involvement of pathways related to protein synthesis and amino acid metabolism suggesting a more limited adaptive response (Supplementary Fig. 8a and 8b).

In summary, unsupervised clustering identified two distinct metabolic response groups that closely resembled the metabolic response of the predefined responder and non-responder group. These findings support the subgroup analysis, confirming distinct FMD-induced metabolic adaptations and highlighting interindividual differences in metabolic flexibility and durability.

## 4 Discussion

Our study demonstrates that periodic fasting (PF) effectively restores metabolic flexibility in individuals with T2D and DN, particularly in individuals showing albuminuria improvement. Specifically, we found that (i) PF enhances metabolic flexibility, marked by increased lipid oxidation and reduced amino acid catabolism, (ii) responders to PF display unique biomarker profiles—such as elevated glycine, serine, and cholesteryl esters—associated with improved albuminuria; and (iii) in contrast to the minimal changes observed in the MD group, PF elicited broader and more sustained metabolic adaptations, emphasizing the unique potential of PF despite interindividual variability of metabolic responses in T2D.

### 4.1 Metabolic flexibility induced by PF

In line with our first aim, we show that PF induced a metabolic shift toward increased lipid oxidation, enhanced utilization of fatty acids as primary energy substrates and reduced reliance on amino acid catabolism. The elevated acetylcarnitine/carnitine ratio (C2/C0) observed in the FMD group suggests enhanced acetyl-CoA generation, likely reflecting either increased β-oxidation or acetyl-CoA accumulation surpassing the capacity of the TCA cycle during fasting [11]. Correspondingly, elevated levels of hydroxybutyrylcarnitine (C4-OH) and acetylcarnitine (C2)—end products of β-oxidation—along with a decreased propionylcarnitine/acetylcarnitine ratio (C3/C2), supports decreased anaplerosis and increased cataplerosis towards gluconeogenesis and sparing muscle protein during fasting. [11,41]. A significant decrease in propionylcarnitine (C3) in non-responders after three months may indicate energy exhaustion and an imbalance in replenishing TCA intermediates lost through cataplerosis [42].

The systemic reduction in circulating triglycerides and concurrent increases in acylcarnitines and cholesteryl esters further reflect the improved lipid mobilization and utilization. Although plasma-level changes only indirectly indicate tissue-specific metabolism, they strongly suggest enhanced mitochondrial adaptation [11,41]. The function of lipid changes in PF-induced metabolic adaptation remains to be clarified. They may reflect adherence to the intervention or serve as substrates for renal metabolism, potentially supporting improved albuminuria. Dysregulated lipid metabolism has been shown to be associated with chronic kidney disease progression via oxidative and inflammatory pathways, though certain lipid metabolites may also help resolve inflammation and promote nephroprotection [43]. Furthermore, increased glycine, methionine, serine, and threonine suggest improved protein conservation and cellular maintenance processes, whereas decreased glutamate, alanine, valine, and proline imply heightened gluconeogenesis, ketogenesis or TCA replenishment, reflecting improvement of metabolic flexibility [42].

Collectively, these coordinated shifts illustrate a beneficial systemic metabolic adaptation towards more efficient energy utilization, potentially, protective against T2D-associated complications such as sarcopenia and nephropathy.

### 4.2 Metabolic signatures linked to favourable albuminuria response

To further understand metabolic signatures linked to favourable PF-induced renal response, we compared responders and non-responders based on albuminuria reduction. Responders exhibited sustained metabolic adjustment, with persistent elevations in acylcarnitines, cholesteryl esters, glycine, and serine, coupled with prolonged decrease in triglycerides and phosphatidylcholines by six months, indicating sustained and efficient adaptation to fasting. While acylcarnitine ratio trends were similar across all groups, responders uniquely exhibited higher absolute C2 levels and unchanged C3, reflecting more robust fatty acid oxidation and reduced amino acid catabolism [11,44].

Responder-specific lipid changes included selective increases in lysophosphatidylcholine C20:4 and cholesteryl esters, possibly indicating protective bioactive lipid signalling pathways [45]. In particular, reduced phosphatidylcholines in responders likely indicate increased membrane remodelling and fatty acid utilization, which could contribute directly or indirectly to obesity and T2D [[46], [47], [48]].

Elevated glycine and serine levels in responders further underscore enhanced metabolic flexibility and improved insulin sensitivity, given prior associations of low plasma glycine and serine levels with insulin resistance and impaired kidney function [[49], [50], [51]]. As kidneys are key sites of serine biosynthesis during fasting, sustained serine elevation in responders highlights its potential role in mediating renal protection [52].

Together, these biomarkers point to improved metabolic flexibility and a more robust adaptation to PF in responders. Responders appeared to adapt more gradually, with sustained lipid mobilization over time. In contrast, non-responders showed early metabolic shifts that did not persist, suggesting limited engagement with PF-induced metabolic programming. These findings suggest that albuminuria improvement may be linked to the ability to maintain a flexible, lipid-based energy metabolism across repeated fasting cycles.

### 4.3 Comparison with mediterranean diet and metabolic heterogeneity

In comparison, the MD group exhibited minimal metabolic alterations, limited predominantly to a mild increase in tryptophan betaine, a metabolite linked to gut microbiota modulation and metabolic health [38,39] – in contrast to extensive, sustained metabolic changes induced by PF. Unsupervised clustering further confirmed distinct metabolic response profiles: Cluster 2, predominantly comprising FMD responders, showed delayed but robust metabolic adaptations in lipid and amino acid pathways, alongside clinical improvements. Cluster 1, (MD participants and FMD non-responders), showed earlier but short-lived metabolic changes. These results support our third aim and highlight metabolic flexibility as a critical determinant of dietary interventions efficacy. Moreover, the clustering approach successfully identified subgroup-specific trajectories that mirrored predefined response groups, underscoring potential clinical utility for future interventions.

The clinical observation of a rebound in albuminuria following cessation of PF [8] coupled with recapitulation of the metabolic shifts reported in this study provides compelling real-world evidence that firmly connects metabolic flexibility to kidney health. These findings highlight not only the importance of maintaining metabolic flexibility over time but also its potential role as both a therapeutic target and a stratification tool. Assessing an individual’s baseline metabolic flexibility prior to dietary interventions could enable tailored nutritional strategies, helping to predict who is mostly likely to benefit – and who may not – from fasting-based interventions. Such an approach would represent a key step toward precision nutrition in T2D, aimed at achieving sustained metabolic and renal benefits while minimizing risk.

Circulating ketone body levels were used as biochemical markers of adherence to the diet intervention and showed a significant increase in the intervention group compared to the control group at both timepoints (Supplementary Table 1), consistent with previous reports [8,10,31,53] and indicative of a successful metabolic switch toward fasting. Importantly, the similar elevations in ketone concentrations observed in both responders and non-responders (Supplementary Table 2) suggest comparable adherence across subgroups. These findings imply that while non-responders exhibited a metabolic response indicative of enhanced ketogenesis during fasting, this level of adaptation may have been insufficient to elicit clinical improvements in albuminuria. Moreover, additional mechanisms relevant to renal recovery might be less responsive to periodic fasting, require a longer intervention duration, or necessitate complementary therapeutic strategies.

This analysis has certain limitations and areas for potential improvement. A primary limitation is the small sample size, which may not capture the full spectrum of variation present in the population and therefore lack generalizability. Nonetheless, the robust consensus clustering algorithm enabled the extraction of meaningful insights from this limited dataset. Although the filtering process reduced the number of samples available for longitudinal analysis in both study groups, these stringent data quality control measures were necessary to ensure data reliability and integrity, minimize potential bias and strengthen the robustness of our findings. We acknowledge this limitation and recommend interpreting the results accordingly, while emphasizing that the applied methodology allowed for meaningful biological insights. Although a higher proportion of responders was observed in the intervention group compared to controls, this finding is descriptive and should be interpreted with caution, as the study was not powered or specifically designed to detect differences in response rates between groups. Similarly, while the observed metabolomic changes are consistent with known fasting-related adaptations, it remains unclear—and subject to future investigation—whether these changes are specific to fasting-mimicking protocols or represent general effects of caloric restriction. Improvements in albuminuria in both groups may reflect general intervention effects, including medical contact and dietary counseling that could have induced some caloric restriction. However, substantial metabolic shifts observed only in the FMD group suggest additional effects of fasting on metabolic flexibility, which may partly explain the variability in albuminuria response between responders and non-responders. While the study is limited by its modest sample size and reliance on plasma-based markers, it provides novel insight into the mechanisms by which periodic fasting may benefit metabolic and renal health. Future studies with larger cohorts and direct assessments of tissue metabolism will be essential to validate these findings, clarify the roles of identified lipid species, and determine how they interact with renal metabolism to influence clinical outcomes. While circulating ketone bodies provided supportive biochemical evidence of adherence, direct adherence measures (e.g., device-based monitoring, continuous blood glucose and ketone monitoring) were not employed in this study. Incorporating more comprehensive adherence measures in future studies would strengthen the interpretation of intervention effects and help disentangle the relationship between metabolic response and clinical outcomes. Additionally, the clustering results were sensitive to the choice of algorithm, suggesting that future studies should explore various clustering methods and incorporate clinical information to identify the most suitable approach. With careful consideration of experimental design and analytical methods, this unsupervised clustering approach could become a valuable tool for researchers and clinicians, ultimately enhancing patient care.

## CRediT authorship contribution statement

**Alba Sulaj:** Writing – review & editing, Writing – original draft, Visualization, Validation, Supervision, Software, Resources, Project administration, Methodology, Investigation, Funding acquisition, Formal analysis, Data curation, Conceptualization. **Phong B.H. Nguyen:** Writing – review & editing, Writing – original draft, Visualization, Validation, Software, Methodology, Formal analysis, Conceptualization. **Gernot Poschet:** Writing – review & editing, Validation, Resources, Methodology. **Elisabeth Kliemank:** Writing – review & editing, Resources, Project administration, Data curation. **Thomas Fleming:** Writing – review & editing, Methodology. **Lea Henke:** Writing – review & editing, Data curation. **Wiebke Neibig:** Writing – review & editing, Data curation. **Stefan Kopf:** Writing – review & editing, Project administration. **Rüdiger Hell:** Writing – review & editing, Resources, Methodology. **Valter D. Longo:** Writing – review & editing, Resources. **Stephan Herzig:** Writing – review & editing, Methodology. **Peter P. Nawroth:** Writing – review & editing, Supervision. **Michael P. Menden:** Writing – review & editing, Writing – original draft, Supervision, Methodology, Investigation, Formal analysis, Conceptualization. **Julia Szendroedi:** Writing – review & editing, Writing – original draft, Supervision, Methodology, Investigation, Formal analysis, Conceptualization.

## Prior presentation

The study was presented at the Annual Meeting of the European Association for the Study of Diabetes (EASD) in Madrid on 10th September 2024.

## Code availability

Source code from the analysis can be downloaded from github: https://github.com/phngbh/FMD.

## Funding

This study was funded by the 10.13039/501100001659Deutsche Forschungsgemeinschaft (DFG, German Research Foundation; project number 236360313–SFB 1118). A.S, P.B.H.N and M.P.M. were supported by the Deutsches Zentrum für Diabetesforschung (DZD; German Center for Diabetes Research). A.S was further supported by the DZD project 82DZD07C2G, by the 10.13039/501100010215Deutsche Diabetes Gesellschaft (DDG, German Diabetes Society) and by the Olympia Morata funding program of the 10.13039/501100010441Medical Faculty Heidelberg.

## Declaration of competing interest

The authors declare the following financial interests/personal relationships which may be considered as potential competing interests: Alba Sulaj reports equipment, drugs, or supplies was provided by L-Nutra Inc. The authors declare that there are no relationships or activities that might bias, or be perceived to bias, their work. L-Nutra, as founder of the fasting-mimicking diet used in this study, has no role in the design or conduct of the study nor in the preparation, review, or approval of the manuscript. V.L. is founder and shareholder of L-Nutra; his shares are destined to the Create Cures Foundation and other charitable and research organizations. If there are other authors, they declare that they have no known competing financial interests or personal relationships that could have appeared to influence the work reported in this paper.

## Data Availability

Data will be made available on request.
