# Supplementary material for: Periodic fasting induced reconstitution of metabolic flexibility improves albuminuria in patients with type 2 diabetes
Source: Mol Metab. 2025 Sep 24;102:102257. doi: 10.1016/j.molmet.2025.102257 (PMC12546980; doi:10.1016/j.molmet.2025.102257)
Supplement: Multimedia component 1 [file mmc1.docx]

**Supplementary Table 1.** Participant characteristics during the study.

|  | **Timepoint** | **Control** | **Intervention** | **^#^P-value** |
| --- | --- | --- | --- | --- |
| Age (years) | Baseline | 67.6 ± 5.6 | 65.3 ± 6.9 | 0.28 |
| Sex females/males | Baseline  3-month  6-month  Follow-up | 5/13  5/13  5/11  5/9 | 6/14  6/14  5/12  4/11 |  |
| BMI (kg/m^2^) | Baseline  3-month  6-month  Follow-up | 30.3 ± 4.5  30.1 ± 4.7  30.2 ± 4.9  29.8 ± 5.0 | 30.9 ± 4.4  **29.1 ± 4.8*****  **27.7 ± 4.3*****  **29.4 ± 4.4*** | 0.69  0.55  0.14  0.82 |
| HbA1c (%) | Baseline  3-month  6-month  Follow-up | 7.7 ± 1.2  **7.2 ± 1.1****  7.7 ± 1.1  8.0 ± 1.5 | 8.0 ± 1.7  **7.2 ± 1.3*****  **6.7 ± 1.3*****  7.6 ± 1.5 | 0.49  0.84  **0.02**  0.49 |
| HbA1c (mmol/mol) | Baseline  3-month  6-month  Follow-up | 60.4 ± 12.5  **54.8 ± 11.9****  61.0 ± 12.5  73.2 ± 44.4 | 63.9 ± 18.3  **55.7 ± 14.0*****  **50.8 ± 12.0*****  59.4 ± 16.6 | 0.52  0.84  **0.02**  0.26 |
| Fasting glucose (mg/dL) | Baseline  3-month  6-month  Follow-up | 158.1 ± 47.3  149.1 ± 48.3  151.2 ± 48.4  167.5 ± 70.5 | 161.4 ± 45.2  146.7 ± 61.7  **137.4 ± 52.2***  151.9 ± 33.8 | 0.83  0.90  0.44  0.43 |
| Serum creatinine (mg/dL) | Baseline  3-month  6-month  Follow-up | 0.9 ± 0.3  0.9 ± 0.3  0.8 ± 0.3  0.8 ± 0.3 | 0.9 ± 0.3  0.9 ± 0.3  0.9 ± 0.3  0.9 ± 0.3 | 0.75  0.70  0.44  0.37 |
| eGFR CKD-EPI creatinine (mL/min/1.73 m2) | Baseline  3-month  6-month  Follow-up | 85.3 ± 17.9  85.0 ± 19.0  86.4 ± 18.7  87.7 ± 16.7 | 84.1 ± 20.0  83.3 ± 18.8  81.8 ± 19.7  82.3 ± 18.7 | 0.85  0.78  0.49  0.40 |
| Urinary albumin-to-creatinine ratio | Baseline  3-month  6-month  Follow-up | 73.6 (33.5; 347.5)  64.8 (17.3; 244.1)  63.1 (15.5; 398.1)  76.0 (17.1; 187.9) | 49.6 (31.0; 152.2)  34.8 (18.2; 83.0)  25.6 (14.3; 57.3)  27.0 (11.8; 160.8) | 0.69  0.25  0.43  0.51 |
| ALT (U/L) | Baseline  3-month  6-month  Follow-up | 31.7 ± 12.3  32.8 ± 18.6  30.4 ± 11.1  30.7 ± 10.6 | 30.7 ± 14.8  34.7 ± 15.4  25.6 ± 7.9  23.3 ± 9.5 | 0.82  0.74  0.16  0.051 |
| AST (U/L) | Baseline  3-month  6-month  Follow-up | 27.9 ± 7.6  31.1 ± 12.9  27.2 ± 6.6  26.3 ± 5.7 | 27.8 ± 15.3  29.9 ± 10.7  25.5 ± 5.8  23.0 ± 6.5 | 0.96  0.75  0.45  0.14 |
| Triglycerides (mg/dL) | Baseline  3-month  6-month  Follow-up | 170.5 (132.0; 261.3)  172.5 (116.3; 240.8)  202.5 (132.3; 285.3)  179.0 (151.0; 229.0) | 161.5 (112.0; 300.3)  133.0 (83.0; 163.3)  112.0 (84.5; 152.5)  159.5 (80.5; 340.5) | 0.75  0.67  **0.004**  0.92 |
| LDL cholesterol (mg/dL) | Baseline  3-month  6-month  Follow-up | 95.9 ± 38.5  90.2 ± 31.9  96.6 ± 33.7  103.8 ± 44.5 | 86.3 ± 38.6  95.2 ± 50.0  87.2 ± 39.6  84.6 ± 37.6 | 0.42  0.72  0.47  0.24 |
| Blood ketones (mmol/L) | Baseline  3-month  6-month  Follow-up | 0.1 ± 0.1  0.2 ± 0.1  0.1 ± 0.1  0.1 ± 0.1 | 0.2 ± 0.2  **0.5 ± 0.4*****  **0.5 ± 0.5***  0.2 **±** 0.1 | 0.33  **0.002**  **0.01**  0.69 |

Data shown as mean ± SD for normally distributed variables, and as median (interquartile range) for non-normally distributed variables. Data distribution tested with D’Agostino and Pearson normality test.

^#^P-value indicates significant difference of parameters between study groups from unpaired two-tailed t-test or Mann-Whitney test.

*P < 0.05, **P < 0.01, ***P<0.001 indicates significant difference of parameters between baseline and respective timepoint within study group from paired two-tailed t-test.

*Abbreviations:* BMI body mass index, eGFR estimated glomerular filtration rate, ALT alanine transaminase, AST aspartate aminotransferase, LDL low-density lipoprotein cholesterol.

**Supplementary Table 2.** Characteristics of responders and non-responders during the study.

|  | **Timepoint** | **Responders** | **Non-Responders** | **^#^P-value** |
| --- | --- | --- | --- | --- |
| Age (years) | Baseline | 67.8 ± 5.2 | 64.3 ± 7.1 | 0.33 |
| Sex females/males | Baseline  3-month  6-month  Follow-up | 4/7  4/7  4/6  3/6 | 2/7  2/7  1/6  1/5 | 0.64 |
| BMI (kg/m^2^) | Baseline  3-month  6-month  Follow-up | 29.2 ± 3.3  **27.0 ± 3.2*****  **26.0 ± 2.9*****  **27.7 ± 2.9*** | 32.0 ± 5.3  **31.7 ± 5.3****  **30.2 ± 4.9****  31.8 ± 5.3 | 0.26  **0.03**  **0.04**  0.07 |
| HbA1c (%) | Baseline  3-month  6-month  Follow-up | 7.7 ± 1.5  **7.0 ± 1.1***  **6.6 ± 1.2***  7.7 ± 1.8 | 8.4 ± 1.9  **7.6 ± 1.5****  **6.7 ± 1.6***  7.4 **±** 1.1 | 0.40  0.33  0.95  0.74 |
| HbA1c (mmol/mol) | Baseline  3-month  6-month  Follow-up | 60.7 ± 16.5  **52.8 ± 11.7***  **49.1 ± 13.0***  60.5 ± 19.3 | 67.9 ± 20.5  **59.1 ± 16.4****  **53.3 ± 10.9***  57.7 ± 12.1 | 0.39  0.33  0.50  0.75 |
| Fasting glucose (mg/dL) | Baseline  3-month  6-month  Follow-up | 159.6 ± 45.4  **125.9 ± 37.0***  **125.6 ± 42.6***  150.7 ± 35.2 | 163.6 ± 47.7  172.1 ± 77.4  154.3 ± 63.1  154.0 ± 34.6 | 0.85  0.10  0.28  0.86 |
| Serum creatinine (mg/dL) | Baseline  3-month  6-month  Follow-up | 0.9 ± 0.3  0.9 ± 0.3  0.9 ± 0.3  0.9 ± 0.3 | 0.9 ± 0.3  0.9 ± 0.2  0.9 ± 0.2  0.9 ± 0.2 | 0.90  0.55  0.98  0.88 |
| eGFR CKD-EPI creatinine (mL/min/1.73 m2) | Baseline  3-month  6-month  Follow-up | 81.9 ± 21.2  77.7 ± 20.4  79.4 ± 22.1  80.6 ± 21.1 | 86.8 ± 19.2  90.0 ± 15.1  85.1 ± 16.6  85.1 ± 15.1 | 0.59  0.15  0.57  0.65 |
| Urinary albumin-to-creatinine ratio | Baseline  3-month  6-month  Follow-up | 51.3 (38.2; 142.49)  **22.8 (14.5; 36.7)****  **23.4 (12.2; 46.7)***  27.4 (11.4; 159.6) | 43.7 (29.2; 338.1)  92.0 (43.5; 666.1)  44.6 (15.3; 1034.7)  25.2 (12.3; 1184.1) | 0.20  **0.03**  0.08  0.14 |
| ALT (U/L) | Baseline  3-month  6-month  Follow-up | 30.7 ± 10.0  33.5 ± 10.7  **25.4 ± 8.3***  23.4 ± 12.0 | 30.6 ± 19.8  36.2 ± 20.5  26.0 ± 8.1  23.2 ± 3.3 | 0.98  0.70  0.88  0.96 |
| AST (U/L) | Baseline  3-month  6-month  Follow-up | 25.5 ± 5.2  27.0 ± 6.2  25.6 ± 6.5  **21.2 ± 6.7**** | 30.6 ± 22.5  33.3 ± 14.2  25.4 ± 5.1  26.0 ± 5.2 | 0.52  0.20  0.95  0.16 |
| Triglycerides (mg/dL) | Baseline  3-month  6-month  Follow-up | 161.0 (106.5; 204.5)  98.0 (72.0; 144.0)  92.5 (76.5; 135.5)  128.5 (70.5; 276.0) | 162.0 (118.0; 360.0)  137.0 (110.5; 309.0)  142.0 (89.0; 208.0)  245.5 (105.3; 396.8) | 0.50  0.09  0.051  0.46 |
| LDL cholesterol (mg/dL) | Baseline  3-month  6-month  Follow-up | 84.1 ± 34.2  82.7 ± 46.0  86.3 ± 49.6  95.3 ± 36.1 | 87.7 ± 43.6  112.4 ± 53.1  88.6 ± 22.3  65.4 ± 35.5 | 0.84  0.21  0.91  0.16 |
| Blood ketones (mmol/L) | Baseline  3-month  6-month  Follow-up | 0.1 ± 0.1  **0.4 ± 0.3***  **0.5 ± 0.4***  0.1 ± 0.1 | 0.2 ± 0.3  **0.6 ± 0.5****  **0.6 ± 0.7***  0.2 ± 0.1 | 0.27  0.52  0.65  0.54 |

Data shown as mean ± SD for normally distributed variables, and as median (interquartile range) for non-normally distributed variables. Data distribution tested with D’Agostino and Pearson normality test.

^#^P-value indicates significant difference of parameters between study groups from unpaired two-tailed t-test or Mann-Whitney test.

*P < 0.05, **P < 0.01, ***P<0.001 indicates significant difference of parameters between baseline and respective timepoint within study group from paired two-tailed t-test.

*Abbreviations:* BMI body mass index, eGFR estimated glomerular filtration rate, ALT alanine transaminase, AST aspartate aminotransferase, LDL low-density lipoprotein cholesterol.

**Supplementary Table 3.** Characteristics of cluster 1 and cluster 2 during the study.

|  | **Timepoint** | **Cluster 1** | **Cluster 2** | **P-value** |
| --- | --- | --- | --- | --- |
| Age (years) | Baseline | 68.0 ± 6.4 | 65.4 ± 5.0 | 0.22 |
| Sex females/males | Baseline  3-month  6-month  Follow-up | 7/13  7/13  7/13  6/12 | 3/8  3/8  3/8  3/8 | 1 |
| BMI (kg/m2) | Baseline  3-month  6-month  Follow-up | 29.5 ± 4.9  **28.7 ± 5.0*****  **28.4 ± 5.1*****  **29.1 ± 5.1**** | 31.2 ± 2.6  **29.7 ± 3.8***  **29.3 ± 4.0***  30.3 ± 3.7 | 0.21  0.59  0.65  0.52 |
| HbA1c (%) | Baseline  3-month  6-month  Follow-up | 7.5 ± 1.2  **7.1 ± 1.2*****  7.4 ± 1.2  7.6 ± 1.2 | 8.1 ± 1.6  **7.0 ± 1.1****  **6.7 ± 1.4****  8.2 ± 2.0 | 0.35  0.78  0.16  0.28 |
| HbA1c (mmol/mol) | Baseline  3-month  6-month  Follow-up | 58.8 ± 13.4  **54.5 ± 13.0*****  57.1 ± 13.6  66.2 ± 38.8 | 66.0 ± 17.6  **53.1 ± 12.4****  **51.8 ± 12.0***  65.9 ± 21.4 | 0.16  0.77  0.29  0.98 |
| Fasting glucose (mg/dL) | Baseline  3-month  6-month  Follow-up | 158.0 ± 52.0  148.9 ± 65.1  155.0 ± 56.4  156.1 ± 48.3 | 160.9 ± 41.1  **130.7 ± 31.4***  **117.9 ± 26.6****  165.6 ± 66.0 | 0.87  0.39  **0.05**  0.65 |
| Serum creatinine (mg/dL) | Baseline  3-month  6-month  Follow-up | 0.9 ± 0.3  0.9 ± 0.3  0.9 ± 0.3  0.9 ± 0.3 | 0.8 ± 0.3  0.8 ± 0.2  0.8 ± 0.3  0.8 ± 0.2 | 0.42  0.49  0.44  0.60 |
| eGFR CKD-EPI creatinine (mL/min/1.73 m2) | Baseline  3-month  6-month  Follow-up | 83.2 ± 19.1  82.0 ± 20.4  82.0 ± 19.4  82.5 ± 18.8 | 91.4 ± 16.0  88.8 ± 15.3  90.4 ± 16.5  89.2 ± 15.3 | 0.22  0.34  0.23  0.32 |
| Urinary albumin-to-creatinine ratio (median [IQR]) | Baseline  3-month  6-month  Follow-up | 47.8 (31.5; 153.1)  42.5 (18.7; 106.9)  52.8 (22.5; 169.6)  25.2 (16.6; 158.3) | 73.7 (37.0; 181.2)  31.2 (15.3; 107.0)  23.2 (10.4; 50.0)  64.9 (11.4; 283.9) | 0.69  0.68  0.85  0.25 |
| ALT (U/L) | Baseline  3-month  6-month  Follow-up | 30.0 ± 10.5  31.5 ± 17.7  27.2 ± 9.6  **26.5 ± 10.0*** | 31.2 ± 12.1  34.1 ± 9.5  29.0 ± 10.1  27.6 ± 12.0 | 0.78  0.65  0.62  0.77 |
| AST (U/L) | Baseline  3-month  6-month  Follow-up | 27.7 ± 7.2  29.0 ± 12.8  26.2 ± 6.8  24.4 ± 5.8* | 27.0 ± 7.0  29.2 ± 6.2  26.6 ± 5.6  25.1 ± 7.3 | 0.81  0.96  0.86  0.76 |
| Triglycerides (mg/dL) (median [IQR]) | Baseline  3-month  6-month  Follow-up | 159.5 (113.5; 178.5)  **136.5 (77.3; 177.8)***  140.5 (78.5; 226.8)  157.5 (78.0; 207.5) | 222.0 (162.5; 467.0)  167.0 (94.0; 194.0)  149.0 (103.0; 215.0)  252.0 (146.0; 405.0) | **0.04**  0.72  0.97  **0.01** |
| LDL cholesterol (mg/dL) | Baseline  3-month  6-month  Follow-up | 93.2 ± 33.6  91.1 ± 30.8  89.6 ± 33.9  89.2 ± 35.5 | 90.5 ± 37.5  100.9 ± 58.6  96.6 ± 44.6  105.1 ± 54.0 | 0.84  0.54  0.62  0.37 |
| Blood ketones (mmol/L) | Baseline  3-month  6-month  Follow-up | 0.1 ± 0.1  **0.3 ± 0.3***  0.3 ± 0.4  0.1 ± 0.1 | 0.2 ± 0.2  **0.4 ± 0.5***  0.3 ± 0.4  0.2 ± 0.1 | 0.69  0.62  0.98  0.20 |

Data shown as mean ± SD for normally distributed variables, and as median (interquartile range) for non-normally distributed variables. Data distribution tested with D’Agostino and Pearson normality test.

^#^P-value indicates significant difference of parameters between study groups from unpaired two-tailed t-test or Mann-Whitney test.

*P < 0.05, **P < 0.01, ***P<0.001 indicates significant difference of parameters between baseline and respective timepoint within study group from paired two-tailed t-test.

*Abbreviations:* BMI body mass index, eGFR estimated glomerular filtration rate, ALT alanine transaminase, AST aspartate aminotransferase, LDL low-density lipoprotein cholesterol.

**Supplementary Figure 1.** Comparison of the metabolite levels at baseline between FMD and MD groups.


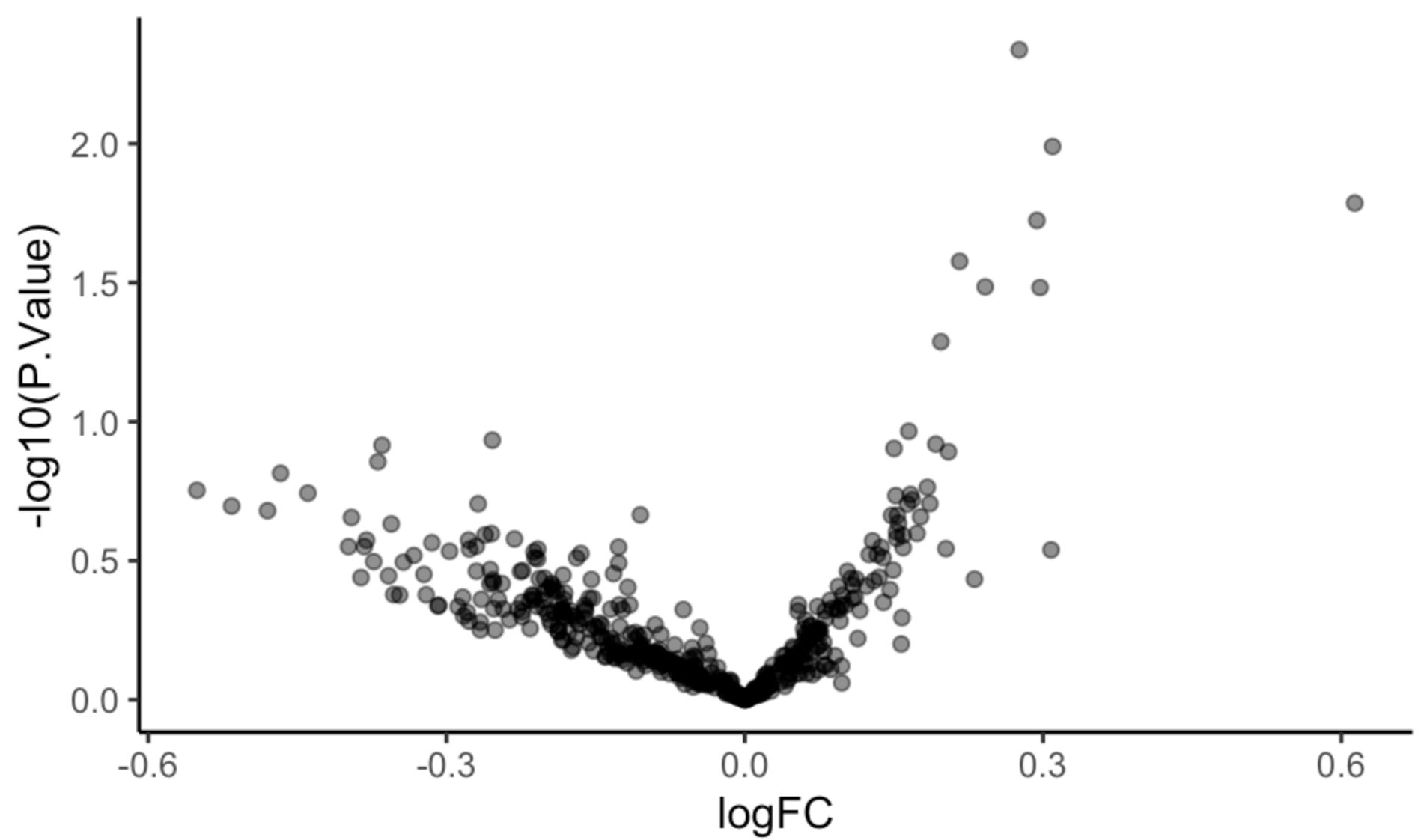


LogFC is the difference of metabolite levels of FMD compared to MD. Significant differences (FDR-adjusted p-values < 0.05) would be colored in red.

**Supplementary Figure 2. Changes in amino acids after three months of intervention.
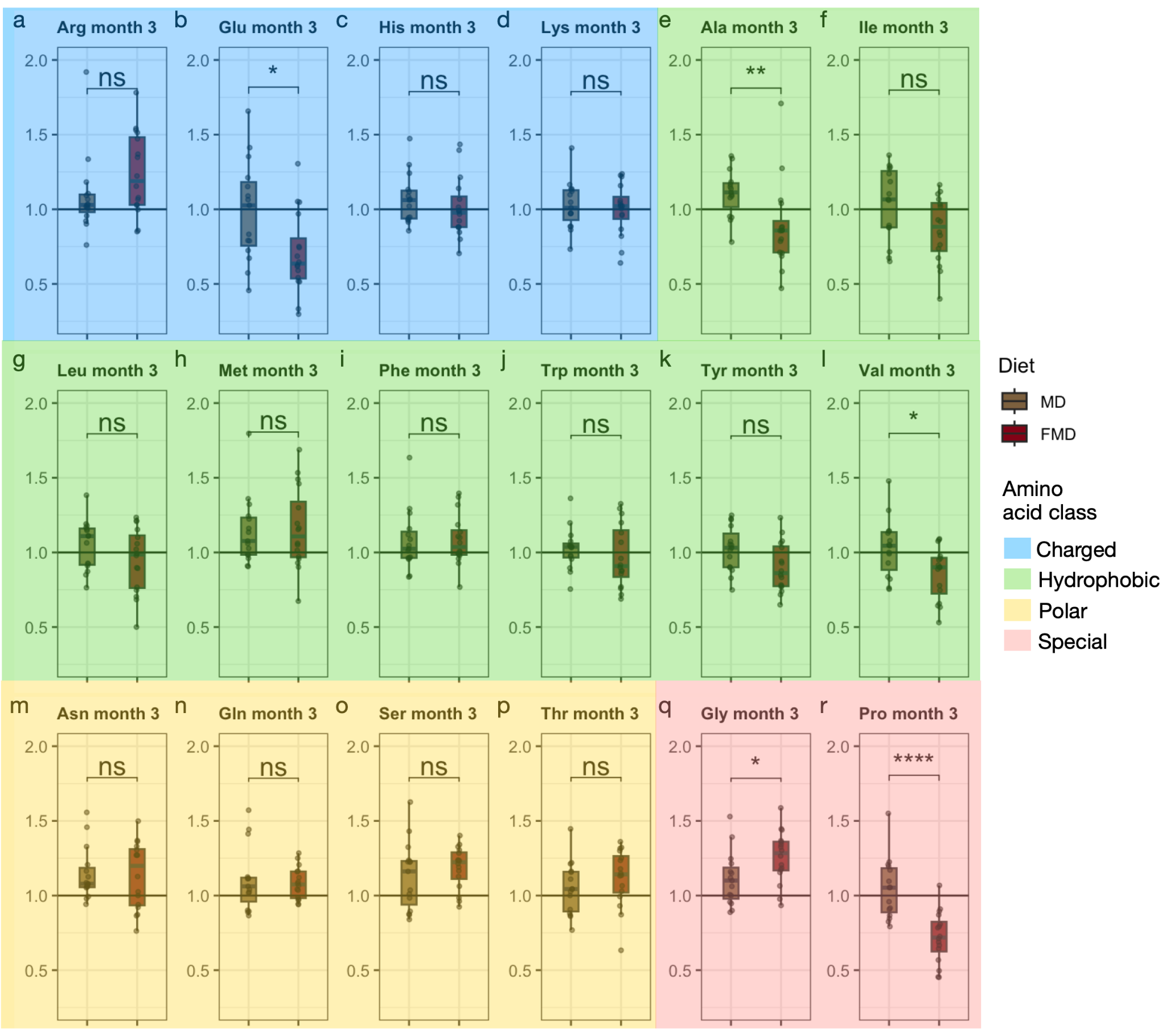
**

**Supplementary Figure 2:** Changes in amino acids after three months of intervention in MD and FMD groups . Here, we are showing absolute change of metabolite normalized to respective baseline value.. Wilcoxon Rank Sum Test was carried out to evaluate the differences between groups, significance level was denoted as “ns” for p > 0.05, * for p <= 0.05, ** for p <= 0.01, *** for p <= 0.001 and **** for p <= 0.0001.

*Abbreviations:* Arg arginine, Glu glutamate, His histidine, Lys lysine, Ala alanine, Ile isoleucine, Leu leucine, Met methionine, Phe phenylalanine, Trp tryptophan, Tyr tyrosine, Val valine, Asn asparagine, Gln Glutamine, Ser serine, Thr threonine, Gly glycine, Pro proline.

**Supplementary Figure 3. Changes in amino acids after six months of intervention.**


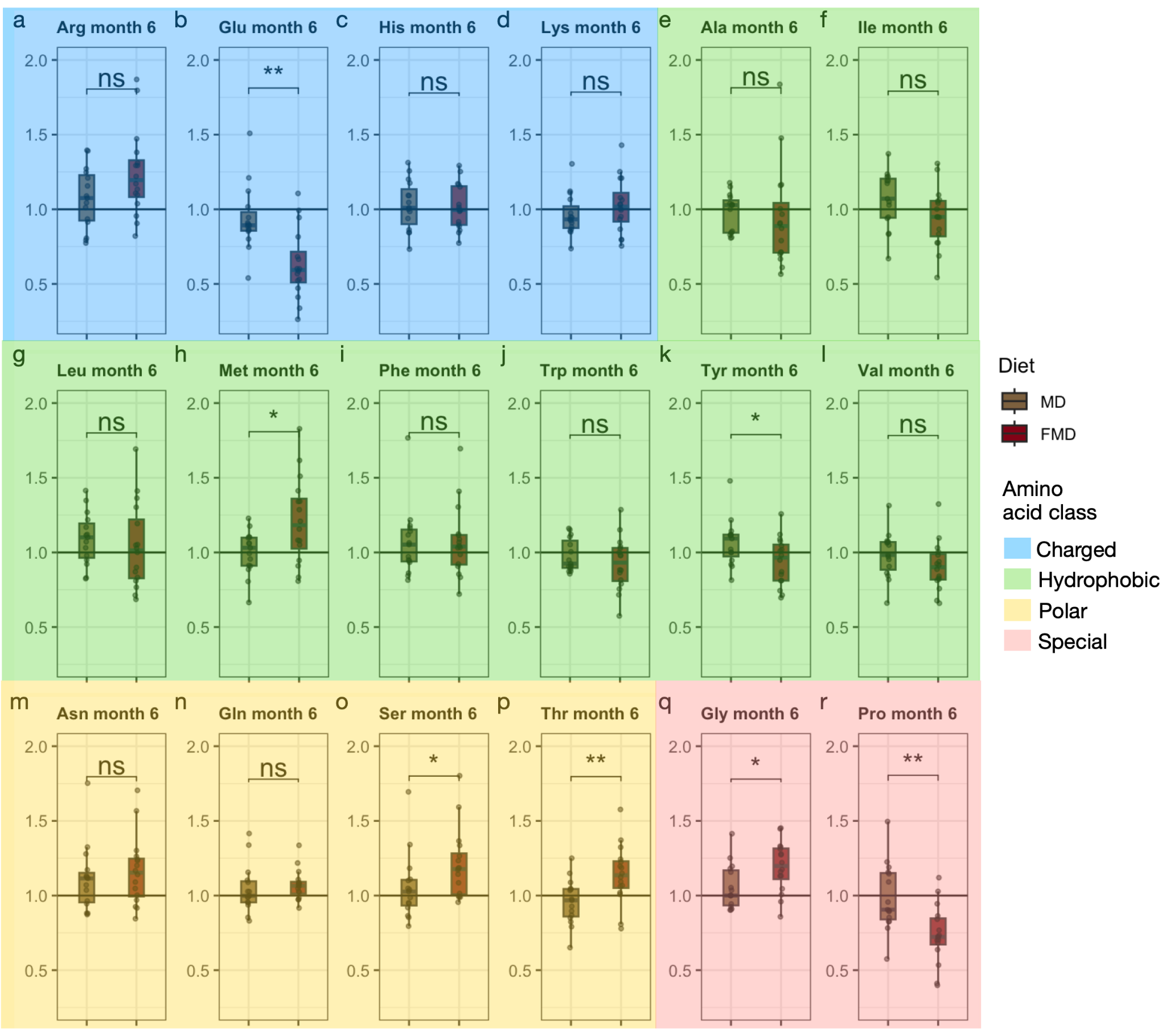


**Supplementary Figure 3:** Changes in amino acids after six months of intervention in MD and FMD groups. Here, we are showing absolute change of metabolite normalized to respective baseline value. Wilcoxon Rank Sum Test was carried out to evaluate the differences between groups, significance level was denoted as “ns” for p > 0.05, * for p <= 0.05, ** for p <= 0.01, *** for p <= 0.001 and **** for p <= 0.0001.

*Abbreviations:* Arg arginine, Glu glutamate, His histidine, Lys lysine, Ala alanine, Ile isoleucine, Leu leucine, Met methionine, Phe phenylalanine, Trp tryptophan, Tyr tyrosine, Val valine, Asn asparagine, Gln Glutamine, Ser serine, Thr threonine, Gly glycine, Pro proline.

**Supplementary Figure 4. Changes in amino acids at follow up.
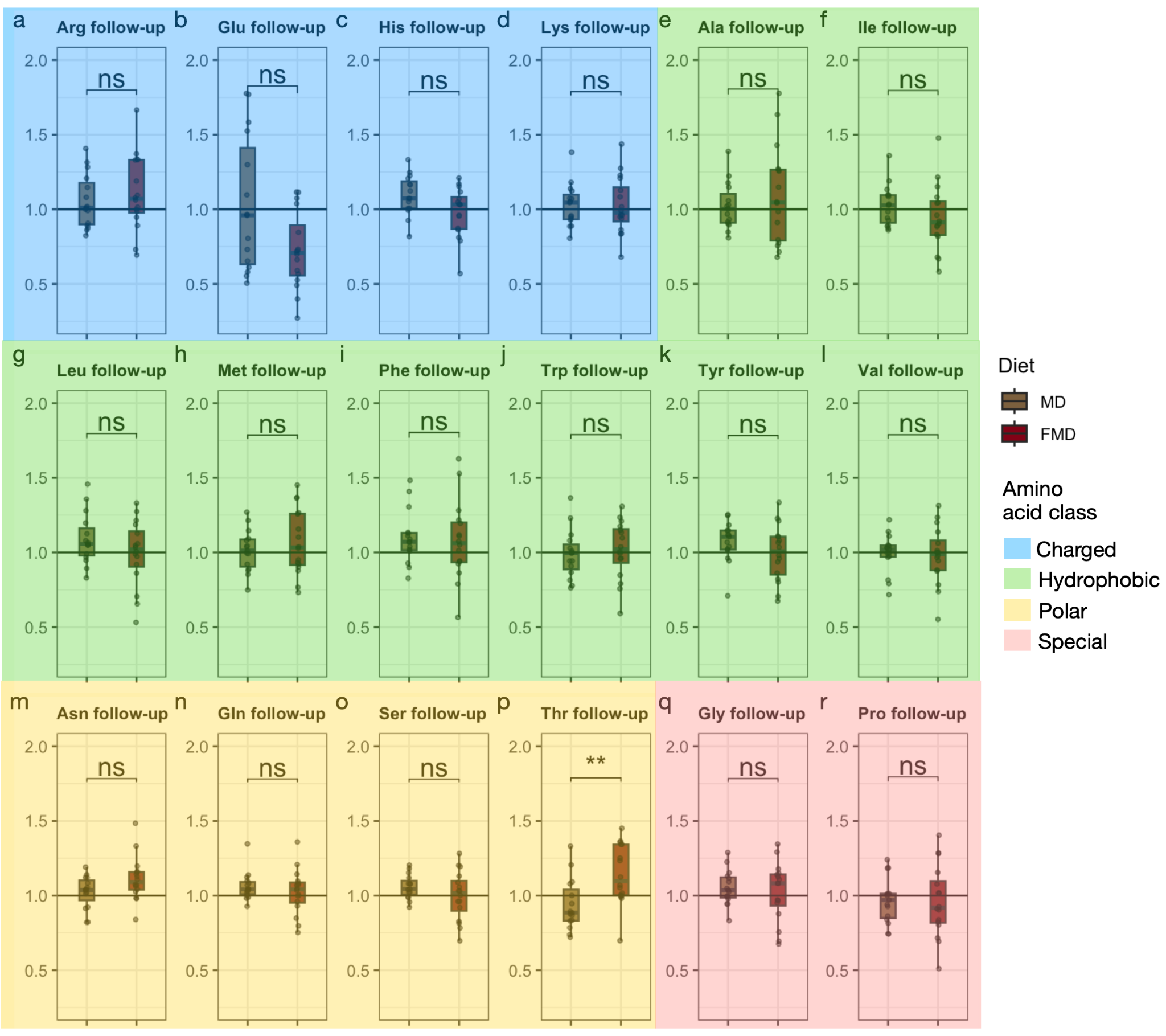
**

**Supplementary Figure 4:** Changes in amino acids at follow up after intervention in MD and FMD groups. Here, we are showing absolute change of metabolite normalized to respective baseline value. Wilcoxon Rank Sum Test was carried out to evaluate the differences between groups, significance level was denoted as “ns” for p > 0.05, * for p <= 0.05, ** for p <= 0.01, *** for p <= 0.001 and **** for p <= 0.0001.

*Abbreviations:* Arg arginine, Glu glutamate, His histidine, Lys lysine, Ala alanine, Ile isoleucine, Leu leucine, Met methionine, Phe phenylalanine, Trp tryptophan, Tyr tyrosine, Val valine, Asn asparagine, Gln Glutamine, Ser serine, Thr threonine, Gly glycine, Pro proline.

**Supplementary Figure 5. Changes in ratios between various acylcarnitines.**

| **a**   | **b**   |
| --- | --- |

**Supplementary Figure 5.** These ratios showed significant differences (based on ANOVA, adjusted p-values < 0.05) among study groups.

*Abbreviations:* C2 acetylcarnitine, C0 carnitine, V0 baseline, V3 and V6 3 months and 6 months after diet intervention, FU follow-up, MD Mediterranean diet, FMD fasting-mimicking diet.

**Supplementary Figure 6. Changes in metabolites in the control group during the study.**

| **a**  **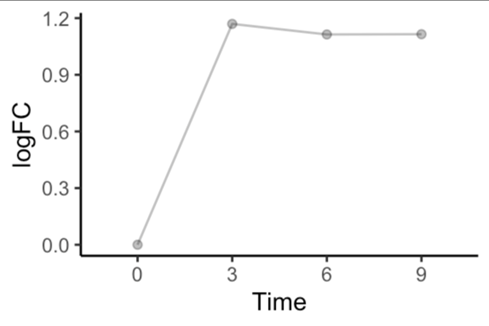** | **b**  **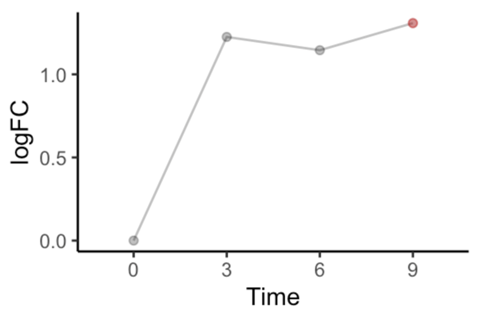** |
| --- | --- |

Longitudinal metabolomic changes in a) control group responder, and b) control group non-responder. LogFC is the log-transformation of the fold change. Significantly differential metabolites are marked in red (tryptophan betaine).

**Supplementary Figure 7. Clinical variables that are significantly changed between the two unsupervised clusters.**


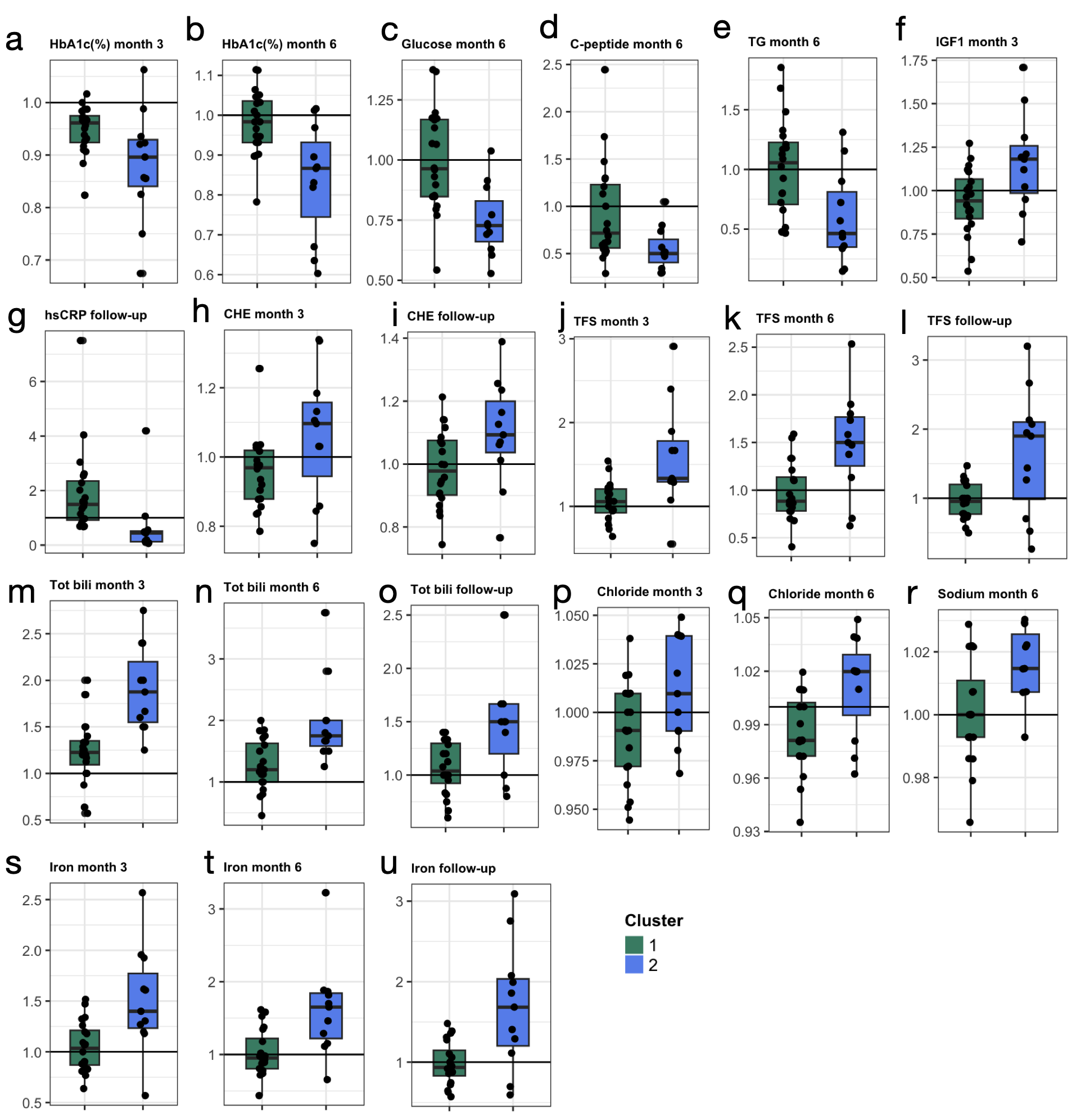


**Supplementary Figure 7.** Clinical variables that are significantly changed between the two unsupervised clusters. These variables showed significant differences (based on ANOVA, adjusted p-values < 0.05) between cluster 1 and cluster 2 during the unsupervised analysis. y-axis shows the values normalized for baseline values, the boxes are median and interquartile range.

*Abbreviations:* TG triglycerides, IGF1 insulin-like growth factor-1, hsCRP high sensitivity C-reactive protein, CHE cholinesterase, TFS transferrin saturation, tot Bili total bilirubin.

**Supplementary Figure 8. Metabolite enrichment pathway analysis in the unsupervised clusters.**


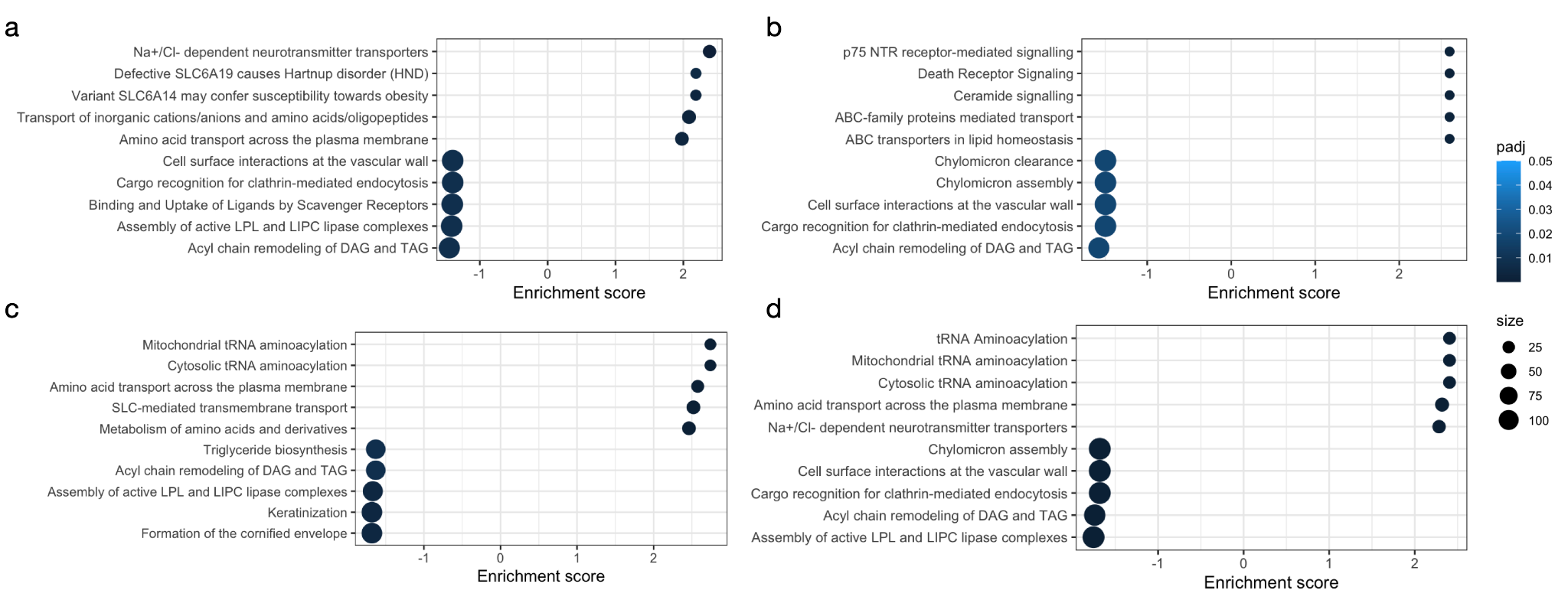


**Supplementary Figure 8.** **Metabolite enrichment pathway analysis in the unsupervised clusters.** Here, highlighted are the top 5 upregulated and top 5 downregulated pathways associated with **a**) Cluster 1 at 3 months, **b**) Cluster 1 at 6 months, **c**) Cluster 2 at 3 months, and **d**) Cluster 2 at 6 months of diet intervention.
